# Characteristics of direct-to-consumer platforms offering erectile dysfunction treatment

**DOI:** 10.1093/sexmed/qfad038

**Published:** 2023-08-02

**Authors:** Sarah M Brink, Teona Iarajuli, David Shin

**Affiliations:** Department of Urology, Hackensack University Medical Center, 360 Essex Street, Suite 403, Hackensack, NJ 07601, United States; Department of Urology, Hackensack Meridian School of Medicine, 123 Metro Boulevard, Suite 4100, Nutley, NJ 07110, United States; Department of Urology, Hackensack University Medical Center, 360 Essex Street, Suite 403, Hackensack, NJ 07601, United States; Department of Urology, Hackensack Meridian School of Medicine, 123 Metro Boulevard, Suite 4100, Nutley, NJ 07110, United States

**Keywords:** direct-to-consumer, telehealth, online platform, erectile dysfunction

## Abstract

**Background:**

Due to the sensitivity and potential embarrassment of discussing erectile dysfunction (ED) in person, men are seeking treatment online.

**Aims:**

We sought to compare offerings of direct-to-consumer (DTC) platforms for ED treatment with respect to consultation, pricing, services, and privacy policy.

**Methods:**

Google was queried to identify DTC platforms offering ED treatment with the keywords: “telehealth erectile dysfunction,” “telemedicine erectile dysfunction,” and “online erectile dysfunction.” Inclusion criteria were as follows: serving a majority of U.S. states, existing online only, providing both the consultation and prescription for phosphodiesterase type 5 inhibitors, and delivering the prescription to the patient.

**Results:**

Fifteen DTC platforms met criteria. Ten provided free consultations; 4 bundled the consultation fee with the first month of the prescription, with 1 of these functioning as a subscription service. Fourteen (93%) relied on online intake forms and 10 (67%) advertised review by the prescriber within 2 business days. Only 4 (27%) platforms explicitly advertised physician-only consults. Direct contact with the prescriber would only occur if needed or if required by state law at 8 (53%) platforms. Purchasing sildenafil and tadalafil was advertised on all platforms. Minimum prices of sildenafil ranged from $0.50 to $35/pill (mean $5.16/pill, median $2.65/pill); tadalafil ranged from $0.50 to $9.80/pill (mean $4.70/pill, median $3.21/pill). In addition to ED therapy, 13 (86%) platforms offered treatment for other men’s health issues. All platforms included a website privacy policy, but only 10 (67%) mentioned Health Insurance Portability and Accountability Act compliance, with 2 of these claiming to not be covered entities.

**Conclusion:**

Although DTC platforms are transparent with phosphodiesterase type 5 inhibitor medication and subscription pricing information, few offer direct contact with a physician to further discuss issues related to ED after completion of the online intake form. For comprehensive evaluation of ED in Health Insurance Portability and Accountability Act–compliant settings, in-person or telemedicine visits should be arranged with men’s physicians.

## Introduction

Within the field of urology, telemedicine has been increasing in usage, catalyzed by the COVID-19 pandemic.^1^ The expansion of telemedicine has the potential to benefit men with erectile dysfunction (ED) who may otherwise choose to defer treatment due to inconvenience, shame, or perceived indiscretion.^2^ Burnett et al^3^ found that ED affects about 18% of the male population of the United States over 20 years of age, with prevalence increasing with age. Multiple online-only telehealth platforms offer services for men’s sexual health. We sought to compare the offerings of U.S.-based direct-to-consumer (DTC) telehealth platforms for ED treatment with respect to consultation, price, services provided, and privacy policy.

## Methods

Google was queried in June 2022 to identify DTC platforms offering ED treatment. Two reviewers identified and analyzed the top 50 results using the keywords “telehealth erectile dysfunction,” “telemedicine erectile dysfunction,” and “online erectile dysfunction.” Inclusion criteria were as follows: platforms must serve a majority of U.S. states, exist online only, provide both the consultation and prescription for phosphodiesterase type 5 (PDE5) inhibitors, and deliver the prescription to the patient. Descriptive analysis of pricing information, physician access, and Health Insurance Portability and Accountability Act (HIPAA) compliance was performed.

## Results

Fifteen DTC platforms met inclusion criteria (Blink Health, BlueChew, GoodRx Care, HendRX, Hims, iLiveActive, K Health, KwikMed, Lemonaid Health, Optum, RedBoxRx, RexMD, Roman, Strut, TIN Rx). Ten (67%) provided free consultations; however, patients were expected to pay monthly or quarterly fees for prescriptions. Four (27%) bundled the consultation fee with the first month of the prescription; one of these functioned as a subscription service with a $10 monthly charge plus monthly refill fee (Table 1).

**Table 1:** Fee schedule.

**Platform**	**Online visit**	**Subscription**	**Bundled appointment and first prescription**
Blink Health	—	$9.95+ price of prescription	—
BlueChew	Free	—	—
GoodRx Care	—	—	$39
HendRX	—	—	$40 for 1 prescription, $50 for prescription and 1 refill, $60 for prescription and 2 refills
Hims	Free	—	—
iLiveActive	NA	NA	NA
K Health	Free	—	—
KwikMed	Free	—	—
Lemonaid Health	Free	—	—
Optum	Free	—	—
RedBoxRx	Free	—	—
RexMD	Free	—	—
Roman	Free	—	—
Strut	Free	—	—
TIN Rx	—	—	$85

Abbreviation: NA, not available.

Fourteen (93%) platforms began the consult with an online form and 10 (67%) platforms advertised review by the prescriber within 2 business days. Four (27%) platforms explicitly advertised physician-only consults, 5 (33%) used the vague term “provider,” and the remaining 6 (40%) advertised either physicians or other prescribers on staff. Direct contact with the prescriber (digital/audio/video) would only occur if needed or if required by state law at 8 (53%) platforms (Table 2).

**Table 2:** Consultation.

**Platform**	**Prescriber**	**Intake**	**Contact with prescriber**	**Review/approval**
Blink Health	Physicians and clinicians	Online form	Licensed doctor follows up with medical evaluation	<24 h
BlueChew	“Provider”	Online form	Phone/video if required by state law	1-2 business days
GoodRx Care	“Provider”	Online form	“Chat within minutes”	NA
HendRX	Physician	Online form	Contact if needed	1-2 business days
Hims	“Provider”	Online form	Phone/video if required by state law	NA
iLiveActive	Physician	Online form	15 minute meeting with physician	NA
K Health	Physician or NP	Online form	“Text with a doctor”	12 h
KwikMed	Physician or NP	Online form	Video/audio/digital if required	24 h
Lemonaid Health	Physician or NP	Online form	Phone/video if required by state law	1 d
Optum	“Provider”	Online form	NA	24-48 h
RedBoxRx	“Provider”	Online form	NA	24 h
RexMD	Physician or NP	Online form	Contact if needed	NA
Roman	Physician or NP	Online form	Phone/video if required by state law	24 h
Strut	Physician	Online form	Contact if needed	1 business day
TIN Rx	Physician	NA	NA	NA

Abbreviations: NA, not available; NP, nurse practitioner.

The ability to purchase sildenafil and tadalafil was advertised on all platforms. Pricing information varied depending on pill strength and quantity ordered on 8 (53%) platforms. Six (40%) platforms only offered pricing per dose without specifying medication strength. Minimum prices of sildenafil ranged from $0.50 to $35/pill (mean $5.16/pill, median $2.65/pill), while tadalafil ranged from $0.50 to $9.80/pill (mean $4.70/pill, median $3.21/pill).

In addition to ED therapy, 13 (86%) platforms offered treatment for other men’s health issues, such as premature ejaculation or hair loss. All platforms included a website privacy policy, but only 10 (67%) mentioned HIPAA compliance, with 2 of these claiming to not be covered entities.

## Discussion

Given the rise in DTC telehealth platforms in the United States, this study chose to focus on U.S.-based DTC platforms providing both the consultation and medication to the patient. Platforms that provided only the consultation and prescription, but not the medication itself, were excluded from this study, given that one of the aims was to evaluate the price of medication from these DTC platforms. It is possible to purchase PDE5 inhibitors, or what are advertised as PDE5 inhibitors, from illegitimate online pharmacies without a prescription. There are also sexual enhancement supplements available online, which are unregulated and often contain unlisted sildenafil as well as potentially dangerous ingredients and contaminants.^4^ Prescription medication can also be obtained from Canada and Mexico. Americans can purchase sildenafil through physical or online Canadian pharmacies; however, these pharmacies require a valid prescription. A prescription from a U.S. physician must be cosigned by a Canadian physician in order for the prescription to be valid in Canada.^5^ Caution should be paid when evaluating online Canadian pharmacies. There are illegitimate pharmacies that do not require a prescription and may be based outside of Canada despite advertising themselves as Canadian.^6^ Sildenafil can be purchased without a prescription in Mexico; however, sildenafil purchased in Mexico is not Food and Drug Administration approved and would be illegal to transport across the U.S. border without a valid prescription.^7^ The DTC platforms highlighted in this article provide convenience and perhaps a perceived cost savings to the patient, which lends to their increasing popularity.

DTC platforms meeting inclusion criteria are upfront with their consultation pricing policies, with patients generally knowing what to expect prior to engaging their services. The vast majority of platforms begin the consult through an online form; however, depending on information provided and state law, there may be no synchronous contact with the prescriber. Patients oftentimes need to ask clarifying and follow-up questions, and open dialogue between the patient and prescriber can lead to more comprehensive care. This lack of synchronous communication, and potentially no communication from prescriber to patient, could create a difficult position for the prescriber insofar as the patient may have filled out the form incorrectly leading to a situation in which PDE5 inhibitors are prescribed when they otherwise would not be.

Relying on self-reported vital signs also presents a potential quagmire and may miss a patient who would otherwise be referred to a primary care physician or cardiologist for further evaluation. According to the American Urological Association Guidelines for Erectile Dysfunction, men should be informed of the association between ED and cardiovascular disease, and appropriate referrals should be made. This study was not designed to identify if men are receiving appropriate referrals through these DTC platforms; however, a previous study by Hsiang et al^8^ examined adherence to the American Urological Association guidelines by online platforms, which showed that the relationship between ED and cardiovascular disease was unlikely to be addressed with patients. This presents an opportunity for these platforms to consider establishing referral lines to qualified specialists for continuation of care.

One of the potential advantages of DTC platforms is transparency with medication pricing. However, a wide and variable range was observed, making it difficult for potential patients to adequately evaluate and compare costs based on strength of medication, prior to engagement with the platform. In contrast, direct interactions at a pharmacy will result in an appropriate determination of insurance coverage and cost for prescribed medications, allowing patients to quickly make informed decisions regarding their treatment options. Because pharmacies can dispense these medications in a timely fashion, patients are not subjected to holdups caused by the necessity of an online review or setbacks associated with shipment of medications, which can further delay access to treatment.

Interestingly, many of these platforms are offering additional men’s health services, such as treatment for premature ejaculation and hair loss, and even general healthcare services for all patients. These platforms are expanding access to care in a convenient format, but there are potential drawbacks. As with ED treatment, these additional services are not covered by medical insurance, relying entirely on out-of-pocket payment. DTC platforms should not be seen as a replacement for a primary care physician or a trusted specialist, as they lack both the continuity of care that comes from an established physician relationship and dialogue between patient and prescriber. For an insured patient, ED treatment may be less expensive overall with their established physician in an in-person or telemedicine format, as has been observed in prior studies with PDE5 inhibitors and testosterone therapy.^9^^,^^10^

The lack of guaranteed privacy with the included DTC platforms is also very concerning, as patients should rightfully expect privacy regarding their health information. The patient who is using a DTC platform for ED treatment may be doing so out of utmost concern for his privacy.^2^ The platforms understand the concern for privacy and generally advertise discreet packaging; however, this does not translate to guaranteed privacy of personal health information. The inclusion of privacy policies on the websites is a standard practice and does not confer any increased security of information over other nontelehealth websites that also collect personal information. The HIPAA defines standards for the protection of individual’s protected health information, by covered entities. As these DTC platforms do not transmit electronic data as defined in the statute, they do not meet the definition of a covered entity (https://www.hhs.gov/hipaa/for-professionals/privacy/laws-regulations/index.html). As such, these platforms are not mandated to secure protected health information, which should be worrisome to anyone who values the privacy of their health information. Physicians, clinics, and hospitals operating in the traditional way are held to HIPAA standards, in contrast to these DTC platforms that are unregulated as a whole.

The proliferation of DTC platforms signals a market shift in what contemporary patients expect. Urologists should heed this signal and consider adopting telemedicine into their practices, if not already incorporated. With suitable resources and office support, telemedicine can be easily incorporated into the clinic schedule. Office-based telemedicine would require only the physician and the appropriate technology, freeing the front desk staff, medical assistants, and schedulers for other tasks. It is easier to offer after-hours appointments, which are convenient to many patients, when only the physician needs to be available. The American Urological Association and the Sexual Medicine Society of North America should consider developing guidelines for urologists in the appropriate practice of telemedicine, ensuring that patients will receive comprehensive HIPAA-compliant evaluations and treatments in the telemedicine setting rather than simply pay-to-play prescriptions. While telemedicine does have limitations compared with in-person appointments, it is also an exciting new frontier in expanding access to urologic care, including sexual medicine and general urology.

## Conclusion

Telehealth platforms are changing the face of healthcare to offer evaluation and treatment of health conditions, including ED, privately from the patient’s own home. Although platforms are transparent with PDE5 inhibitor medication and subscription pricing information, very few offer direct contact with a physician to further discuss issues related to ED after completion of the online intake form. For comprehensive evaluations of ED in HIPAA compliant settings, formal in-person or telemedicine visits should be arranged with men’s primary physicians and/or urologists. Urologists should strongly consider adopting telemedicine into their practices to further expand access to care for these men.

## Author Contributions

S.M.B. (Conceptualization, Data Curation, Formal Analysis, Methodology, Project Administration, Validation, Writing - Original Draft, Writing - Review & Editing); T.I. (Data Curation), D.S. (Conceptualization, Formal Analysis, Methodology, Project Administration, Supervision, Validation, Writing - Review & Editing).

## Funding

This research did not receive any specific grant from funding agencies in the public, commercial, or not-for-profit sectors.

*Conflict of interest*: None.

## Data Availability

The data underlying this article are available in the article and will be shared on reasonable request to the corresponding author.
